# Association of laxatives use with incident dementia and modifying effect of genetic susceptibility: a population-based cohort study with propensity score matching

**DOI:** 10.1186/s12877-023-03854-w

**Published:** 2023-03-04

**Authors:** Jiangtao Feng, Nan Zheng, Xutong Fan, Shu Li, Yuhan Jiang, Xianfu Yi, Hongxi Yang

**Affiliations:** 1 Department of Orthopedics, Tianjin NanKai Hospital, Tianjin, 300100 China; 2 Department of Network Security and Informatization, Tianjin Medical University, Tianjin, 300070 China; 3 Department of Bioinformatics, School of Basic Medical Sciences, Tianjin Medical University, Qixiangtai Road 22, Heping District, Tianjin, 300070 China; 4 School of Management, Tianjin University of Traditional Chinese Medicine, Tianjin, 301617 China; 5 Department of Pharmacology, Tianjin Key Laboratory of Inflammation Biology, School of Basic Medical Sciences, Tianjin Medical University, Qixiangtai Road 22, Heping District, Tianjin, 300070 China

**Keywords:** Laxatives, Genetic risk, Dementia, UK Biobank

## Abstract

**Background:**

Constipation was associated with incidence of dementia and cognitive decline. Laxatives are the mainstay of constipation management and are commonly used among older populations for both treatment and prevention of constipation. However, the association between use of laxatives and incident dementia, and whether laxatives use may modify the effect of genetic predisposition on dementia remains unclear.

**Methods:**

We applied 1:3 propensity score matching to balance the baseline characteristics of the laxative users versus non-users and to reduce potential confounders using multi-variates adjusted Cox hazards regression models. We categorized genetic risk into three groups (low, middle, and high) through a genetic risk score of common genetic variants. Information on laxatives use was assessed at baseline and categories into four varieties, including bulk forming laxatives, softeners and emollients, osmotic laxatives, and stimulant laxatives.

**Results:**

Of 486,994 participants, there were 14,422 laxatives users in UK Biobank. After propensity score matching, participants with use of laxatives (*n* = 14,422) and matched non-laxative (*n* = 43,266) exposed individuals were enrolled. Over follow-up to 15 years, there were 1377 participants developed dementia (539 for Alzheimer’s disease, and 343 for vascular dementia). The use of laxatives had greater risk of dementia (HR, 1.72; 95% CI:1.54–1.92), Alzheimer’s disease (HR, 1.36; 95% CI: 1.13–1.63), and vascular dementia (HR, 1.53; 95% CI: 1.23–1.92). Compared to non-laxative exposed participants, those with use of softeners and emollients drugs, stimulant laxatives, and osmotic laxatives were associated with 96% (HR, 1.96; 95 CI: 1.23–3.12; *P* = 0.005), 80% (HR, 1.80; 95% CI: 1.37–2.37; *P* < 0.001), and 107% (HR, 2.07; 95% CI: 1.47–2.92; *P* < 0.001) higher risk of developed incident dementia, respectively. In joint effect analysis, compared to participants with low/middle genetic susceptibility and non-laxatives use, the HR (95% CIs) of dementia was 4.10 (3.49–4.81) for those with high genetic susceptibility plus use of laxatives. There was an additive interaction between laxatives use and genetic susceptibility on dementia (RERI: 0.736, 95% CI: 0.127 to 1.246; AP: 0.180, 95% CI: 0.047 to 0.312).

**Conclusions:**

Use of laxatives was associated with higher risk of dementia and modify the effect of genetic susceptibility on dementia. Our findings suggested that attention should be paid to the relationship between laxatives use and dementia, especially in people at high genetic susceptibility.

**Supplementary Information:**

The online version contains supplementary material available at 10.1186/s12877-023-03854-w.

## Introduction

Constipation is one of the most common gastrointestinal disorders, and it is estimated that up to one third of people over 65 years are affected by constipation during their lifetime [1]. Laxatives are the mainstay of constipation management and are commonly used by old people for both treatment and prevention of constipation [2]. Laxatives may cause electrolyte and serum potassium imbalance that negatively affect the renal and cardiovascular systems and can be life threatening [3]. Currently, laxatives being freely available without prescription in many countries, while evidence regarding the effectiveness and safety of laxatives in older populations is lacking.

Constipation and dementia have similar epidemiological characteristics and are often comorbidities [4–6]. Longitudinal or multicenter studies have shown that constipation was associated with incidence of dementia and cognitive decline [7–9]. Laxatives may be used for both treatment and prevention of constipation. However, the evidence of whether laxatives use could reduce the risk of dementia through treating constipation was limited. Up to now, only one retrospective cohort study in Taiwan found that patients who used magnesium oxide, a saline laxative, had a decreased risk of developing dementia [10]. Therefore, we hypothesis that the use of laxatives may reduce the risk of dementia through treating constipation. While there are a wide range of laxative types, such as bulk forming laxatives, softeners and emollients, stimulant laxatives, osmotic laxatives, prokinetic laxatives, and secretory laxatives [1]. In fact, different types of laxatives may distinct in mechanisms and efficacy profiles. Effective laxatives are available to manage constipation and its subsequent comorbidities. Thus, choice of laxative for management of constipation to decrease the subsequent unhealth conditions in older people should be personalized [11]. However, the association between the different types of laxatives and the risk of dementia through treating constipation remains unclear. Therefore, we hypotheses that different types of laxatives may play distinct role on the incidence of dementia risk through treating constipation.

It is becoming very evident that dementia is a result of the interaction between genetics and the environmental factors. Dementia is of highly heritable [12], and emerging evidence demonstrated that the genetic basis of dementia is polygenic. To date, over 20 genetic variants that associated with dementia were identified through genome-wide association studies (GWASs) [13–15]. An aggregated measure of polygenetic risk for dementia could be derived from aggregating multiple risk alleles. Epidemiological evidence has pointed to significant interactions between genetic susceptibility and environment factors in the context of dementia risk [16–19]. But whether there was an interaction between laxatives use and genetic susceptibility that associated with dementia remain largely unknown. Therefore, we aimed to 1) investigate the association of laxatives use with incident dementia, 2) examine the association between laxative types and incidence of dementia, and 3) quantify the combined association of laxatives use and genetic predisposition with dementia incidence in UK Biobank cohort.

## Methods

### Study design and population

The study conducted from UK Biobank, a population-based cohort, recruited 502,412 participants (aged 37–73 years) across the UK between 2006 and 2010 and were followed up to 2021 [20]. Participants provided completed touch-screen questionnaires, biological samples, and physical examination. We excluded participants with self-reported prevalent dementia or who had a diagnosis of dementia identified in medical records. The present study analyzed data from 486,994 participants. Informed consent was signed by all participants before the data collection. In addition, only data was analyzed and participants were not directly involved.

### Data collection

Body mass index (BMI) was determined through physical measurement, and data on sex, age, ethnicity, education level, employment, socioeconomic status, drinking, food, and physical activity were collected through touchscreen questionnaires and interviews. Ethnicity was categorized as White, Black, Asian and other. Education level was classified as either college/university or other (vocational, lower secondary, or upper secondary). Employment status was categorized as employed versus unemployed. Townsend deprivation index (TDI) was used to define socioeconomic level [21], and classified as low, middle, or high according to tertiles [22]. Non-smokers and smokers (former or current smokers) were the two categories for smoking status. The threshold for excessive alcohol consumption was set at > 14 g per day for female and > 28 g per day for male [23]. Less than 10 MET hours per week of physical activity was classified as low, 10 to 49.9 MET hours per week as moderate, and more than 50 MET hours per week as high [24]. At least three of these five regularly consumed food groups must be consumed in sufficient amounts to constitute a healthy diet (Vegetables ≥3 servings/day; Fruits ≥3 servings/day; Unprocessed red meats ≤1.5 servings/week; Processed meats ≤1 serving/week; Fish ≥2 servings/week) [25]. BMI was grouped as < 25, 25 to 30, and ≥ 30 kg/m^2^. Heart disease and stroke was diagnosed according to self-reported and medical records. Depression symptoms were assessed using the Patient Health Queationanaie-4 (PHQ-4), self-reported, or medical records. Diabetes was ascertained based on self-reported, medical records, and using anti-diabetic agents.

### Assessment of dementia

All-cause dementia, Alzheimer’s disease, and vascular dementia were ascertained based on a self-reported diagnosis of dementia, hospital admission (hospital primary and secondary), and death records (death primary and contributory): dementia (Data-Field 42,018 and 42,019), Alzheimer’s disease (Data-Field 42,020 and 42,021), and vascular dementia (Data-Field 42,022 and 42,023). Codes used in the UK Biobank study to identify dementia cases were shown in the Supplementary Table 1.

### Assessment use of laxatives

Through a touchscreen questionnaire, we collected information on use of laxatives at baseline. Participants were asked “Do you regularly take any of the following? (Medication for pain relief, constipation, heartburn)”. If they answered positively for “Laxatives (e.g., Dulcolax, Senokot)”, they were defined as laxatives users. Besides, details about medication types were extracted from the UK Biobank, which was obtained through a verbal interview by a trained nurse on prescription medications. Short-term medications were not included. We searched all the classes of laxative drugs (Supplementary Table 2) and categories the laxatives into four varieties according to their effects on constipation, including softeners and emollients, bulk forming laxatives, osmotic laxatives, stimulant laxatives. Furthermore, we explored the association between detail dosage of laxatives and the risk of incident dementia. Primary care data for about 230,000 UK Biobank participants was made available in 2019. This dataset contains coded prescribed medications (including prescription date, drug code and, where available, drug name and quantity). When considered the sufficient cases for laxative use and non-use groups, we extracted the three most commonly used laxative drugs through prescription medications, including senna, lactulose, and docusate. We divided the total dosage of each laxative into three groups according to the median: non-laxative use, low (less than the median), and high (more than the median).

### Assessment of polygenic risk score

Based on 29 independent single nucleotide polymorphisms (SNPs) associated with dementia, we calculated the weighted polygenic risk score (PRS) for dementia. The SNPs selected are listed in Supplementary Table 3. Briefly, the effect size of each SNP and dementia was obtained from previous GWAS studies [14, 15, 26–28]. A PRS at the individual level was then calculated by weighting each SNP’s risk alleles (0, 1, or 2) with its effect size using the “-score” command in PLINK. According to the tertile of their Z-standardized PRS, individuals were categories as low, middle, or high genetic predisposition (Supplementary Methods).

### Statistical analyses

We applied 1:3 nearest-neighbor propensity score matching (PSM) to balance the baseline characteristics of the laxative users versus non-users and to reduce potential confounders using a Cox hazards regression model with adjustment for the following: sex, age, ethnicity, socioeconomic level, education attainment, employment status, smoking, alcohol use, physical activity, diet, BMI, heart disease, stroke, hypertension, diabetes, depression, constipation, and cholesterol. If data was missing for a covariate, we used multiple imputations based on five replications and utilized a chained-equation method to account for the missing data [29]. Participants who used laxatives were defined as the exposure group. The follow-up time was determined as the period from the initial evaluation to the occurrence of the first dementia-related incident, death, or November 31, 2021, whichever came first. The proportional hazards assumptions for the Cox model were tested using the Schoenfeld residuals method, and no violations of the assumption were observed (Supplementary Table 4). The balances of matched covariates were evaluated by standardized mean differences (SMD), and less than 10% of differences were considered matched sufficiently. The SMD both before and after matching are displayed in Supplementary Fig. 1. To assess the validity of the study, further analyses were used. We conducted secondary analysis using crude analysis, covariates-adjusted Cox regression, and inverse probability of treatment weighting (IPTW). The Kaplan–Meier survival curves were illustrated to reveal the risk of incident dementia in genetic risk tertile groups, and non-parametric log-rank test was used to compare the curves. The incidence rates of dementia per 1000 person-years were calculated for laxative use and non-use groups.

To evaluate the combined effects of laxative use and genetic predisposition on dementia, dummy variables were developed based on joint exposures to both factors. We performed stratification analysis to assess whether the association of laxative use and dementia risk varied by sex, age, ethnicity, socioeconomic level, education attainment, employment status, smoking, alcohol use, physical activity, diet, BMI, heart disease, stroke, hypertension, diabetes, depression, and constipation. By comparing models with and without a cross-product term, the likelihood ratio test was used to examine the interactions. Additional analyses were conducted to assess the reliable of our study. The main analyses were repeated in a sample that excluded participants with dementia or who died during the first 2 years of follow-up. Additionally, we further explored the association of laxatives use with incidence of dementia when restricting to 0–5, 6–10, and 10+ years of follow-up. Furthermore, we excluded participants who had pre-existing disease, such as heart disease, diabetes, and depression at baseline to evaluate the robust of our study. Finally, we assessed the competing risk of non-dementia death on the association between the use of laxatives and dementia risk using the subdistribution method proposed by Fine and Grey [30]. All analyses were performed using R (version 4.2.0). Statistical significance was defined as two-sided *P*-values< 0.05.

## Results

### Baseline characteristics

Of the 486,993 participants, the mean age was 56.5 (SD: 8.1) years, 222,925 (45.8%) were men, and 14,422 with use of laxatives. During a median follow-up of 12.8 years (IQR, 12.1 to 13.4), 7337 participants developed dementia, 3074 participants developed Alzheimer’s disease, and 1641 participants developed vascular dementia. Table 1 shows the baseline characteristics of the entire cohort. Participants with history of laxatives usage were more like to be older, women, current smokers, excessive alcohol consumption, physical inactive, unhealthy diet, had lower socioeconomic status and education level, with higher rates of comorbidities (heart disease, stroke, hypertension, diabetes, and depression).

**Table 1:** Baseline characteristics before and after propensity score matched (PSM) according to use of laxatives in the UK Biobank cohort

Characteristic	Entire cohort			After PSM		
	None (*n* = 472,571)	Laxatives users (*n* = 14,422)	SMD	Control (*n* = 43,266)	Laxatives users (*n* = 14,422)	SMD
Age, mean (SD), year	56.48 (8.10)	58.08 (7.86)	0.201	58.06 (7.79)	58.08 (7.86)	0.003
Male, n (%)	219,738 (46.5)	3188 (22.1)	0.532	8976 (20.7)	3188 (22.1)	0.033
Ethnicity, n (%)			0.082			0.006
White	445,598 (94.3)	13,347 (92.5)		40,100 (92.7)	13,347 (92.5)	
Black	9128 (1.9)	281 (1.9)		837 (1.9)	281 (1.9)	
Asian and other	17,845 (3.8)	794 (5.5)		2329 (5.4)	794 (5.5)	
Socioeconomic status, n (%)			0.142			0.003
High	158,886 (33.6)	4239 (29.4)		12,741 (29.4)	4239 (29.4)	
Middle	157,931 (33.4)	4459 (30.9)		13,412 (31.0)	4459 (30.9)	
Low	155,754 (33.0)	5724 (39.7)		17,113 (39.6)	5724 (39.7)	
Education level, n (%)			0.226			0.010
College or University	155,825 (33.0)	3304 (22.9)		10,087 (23.3)	3304 (22.9)	
Other	316,746 (67.0)	11,118 (77.1)		33,179 (76.7)	11,118 (77.1)	
Current employment, n (%)			0.360			0.012
Employed	272,904 (57.7)	5772 (40.0)		17,570 (40.6)	5772 (40.0)	
Unemployed	199,667 (42.3)	8650 (60.0)		25,698 (59.4)	8650 (60.0)	
Smoking status, n (%)			0.097			0.013
Non-smoking	259,320 (54.9)	7214 (50.0)		22,420 (50.4)	7214 (50.0)	
Smoking	213,251 (45.1)	7208 (50.0)		21,332 (49.3)	7208 (50.0)	
Alcohol consumption, n (%)			0.119			0.010
Excessive	206,246 (43.6)	7150 (49.6)		21,231 (49.1)	7150 (49.6)	
Moderate	266,325 (56.4)	7272 (50.4)		22,035 (50.9)	7272 (50.4)	
Physical activity, n (%)			0.123			0.011
Low	87,577 (18.5)	3391 (23.5)		9976 (23.1)	3391 (23.5)	
Middle	238,339 (50.4)	6791 (47.1)		20,527 (47.4)	6791 (47.1)	
High	146,655 (31.0)	4240 (29.4)		12,763 (29.5)	4240 (29.4)	
Healthy diet, n (%)	210,512 (44.5)	5923 (41.1)	0.070	17,577 (40.6)	5923 (41.1)	0.009
BMI (kg/m^2^)	27.42 (4.77)	27.67 (5.29)	0.049	27.66 (5.24)	27.67 (5.29)	0.001
Heart disease, n (%)	20,151 (4.3)	1080 (7.5)	0.137	3218 (7.4)	1080 (7.5)	0.002
Stroke, n (%)	7605 (1.6)	516 (3.6)	0.124	1470 (3.4)	516 (3.6)	0.010
Diabetes, n (%)	24,452 (5.2)	999 (6.9)	0.074	3060 (7.1)	999 (6.9)	0.006
Hypertension, n (%)	184,713 (39.1)	5521 (38.3)	0.017	16,461 (38.0)	5521 (38.3)	0.005
Depression, n (%)	27,927 (5.9)	2000 (13.9)	0.269	5629 (13.0)	2000 (13.9)	0.025
Constipation, n (%)	3702 (0.78)	873 (6.05)	0.293	2194 (5.08)	873 (6.05)	0.038
Cholesterol, mean (SD), mmol/l	5.69 (1.12)	5.68 (1.17)	0.012	5.68 (1.15)	5.68 (1.17)	0.002
Genetic risk, categories			0.006			0.006
Low	157,536 (33.3)	4829 (33.5)		14,403 (33.3)	4829 (33.5)	
Middle	157,557 (33.3)	4769 (33.1)		14,433 (33.4)	4769 (33.1)	
High	157,478 (33.3)	4824 (33.4)		14,430 (33.4)	4824 (33.4)	

After 1:3 nearest-neighbor propensity score matching, participants with use of laxatives (*n* = 14,422) and matched non-laxative (*n* = 43,266) exposed individuals were enrolled. No major imbalances in the covariates were observed when evaluated using SMD within groups in the propensity-matched cohort (Table 1, all SMD < 0.05). Over follow-up to 15 years (median: 12.7, IQR: 12.0 to 13.4), of 59,364 participants, there were 1377 participants developed dementia (cumulative incidence rate were 3.5% for laxatives users versus 2.0% for non-laxatives users), 539 participants developed Alzheimer’s disease, and 343 participants developed vascular dementia.

### Association between genetic susceptibility and dementia risk

In multivariate adjusted models, compared to individuals with a low genetic susceptibility, the HRs and 95% CIs of dementia were 1.29 (1.21–1.38) for those with a middle genetic susceptibility and 2.76 (2.60–2.93) for those with a high genetic susceptibility (Supplementary Table 5). Furthermore, the Kaplan-Meier survival curves showed that higher genetic susceptibility was correlated to higher cumulative incidence of dementia (Supplementary Fig. 2). Meanwhile, participants with high genetic susceptibility had greater risk of Alzheimer’s disease (HR, 4.11; 95% CI: 3.71–4.54) and vascular dementia (HR, 2.43; 95% CI: 2.15–2.75), compared to participants with low genetic susceptibility.

### Association between the use of laxatives and dementia risk

**Fig. 1:**
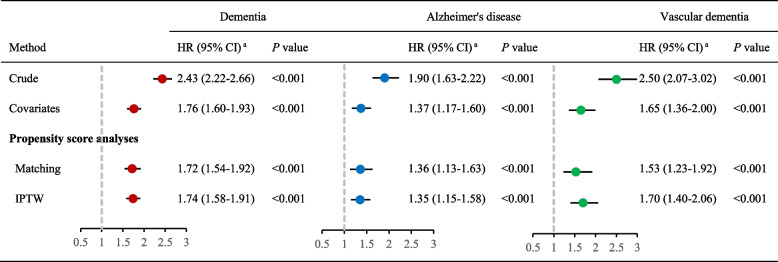
Propensity score matched (PSM) association of laxatives use with incidence of dementia. ^a^ Covariates-adjusted models, propensity-score match models, and IPTW models were adjusted for sex, age, ethnicity, socioeconomic status, education attainment, current employment status, smoking status, alcohol consumption, physical activity, diet, BMI, heart disease, stroke, diabetes, hypertension, depression, cholesterol levels, constipation, and genetic risk. CI, confidence interval; HR, hazard ratio; IPTW, Inverse Probability of Treatment Weighting

In propensity-score match models, the use of laxatives was positively associated with dementia (HR, 1.72; 95% CI:1.54–1.92), Alzheimer’s disease (HR, 1.36; 95% CI: 1.13–1.63), and vascular dementia (HR, 1.53; 95% CI: 1.23–1.92). In Inverse Probability of Treatment Weighting models, compared to non-laxative exposed participants, those with use of laxatives were associated with higher risk of dementia (HR, 1.74; 95% CI: 1.58–1.91), Alzheimer’s disease (HR, 1.35; 95% CI: 1.15–1.58), and vascular dementia (HR, 1.70; 95% CI: 1.40–2.06). Similar, in covariates-adjusted models, the use of laxatives had greater risk of dementia, Alzheimer’s disease, and vascular dementia (All *P* < 0.001; Fig. 1). The cumulative incidence rate of dementia per 1000 person-years was 2.75 for participants who used laxatives and 1.60 for those who did not use laxatives (Supplementary Table 6).

### Association between major type of laxatives and dementia risk

In multi-adjusted Cox regression models, we found that softeners and emollients drugs, stimulant laxatives, and osmotic laxatives were positively associated with dementia, Alzheimer’s disease, and vascular dementia risk, but bulk forming laxatives was not associated with the outcomes (Table 2). Compared to non-laxative exposed participants, those with use of softeners and emollients drugs, stimulant laxatives, and osmotic laxatives were associated with 96% (HR, 1.96; 95 CI: 1.23–3.12; *P* = 0.005), 80% (HR, 1.80; 95% CI: 1.37–2.37; *P* < 0.001), and 107% (HR, 2.07; 95% CI: 1.47–2.92; *P* < 0.001) higher risk of developed incident dementia, respectively. Additionally, bulk forming laxatives, softeners and emollients, stimulant laxatives, and osmotic laxatives were not associated Alzheimer’s disease. Osmotic laxatives were associated with 2.34-folds higher risk of vascular dementia (HR, 2.34; 95% CI: 1.28–4.31; *P* = 0.006).

**Table 2:** Propensity score matched (PSM) association of laxative types with incidence of dementia

Laxative types	Users	Non-laxatives users	Dementia		Alzheimer’s disease		Vascular dementia	
			HR (95% CI) ^a^	*P* value	HR (95% CI)	*P* value	HR (95% CI) ^a^	*P* value
Bulk forming laxatives	289	1.00 (ref.)	1.16 (0.64–2.10)	0.621	1.30 (0.54–3.14)	0.565	1.94 (0.80–4.72)	0.143
Softeners and emollients	322	1.00 (ref.)	1.96 (1.23–3.12)	0.005	1.97 (0.93–4.16)	0.077	1.10 (0.35–3.45)	0.867
Stimulant laxatives	1220	1.00 (ref.)	1.80 (1.37–2.37)	< 0.001	1.12 (0.66–1.92)	0.673	1.16 (0.61–2.19)	0.649
Osmotic laxatives	549	1.00 (ref.)	2.07 (1.47–2.92)	< 0.001	1.63 (0.89–2.98)	0.110	2.34 (1.28–4.31)	0.006

^a^ Models were adjusted for sex, age, ethnicity, socioeconomic status, education attainment, current employment status, smoking status, alcohol consumption, physical activity, diet, BMI, heart disease, stroke, diabetes, hypertension, depression, cholesterol levels, constipation, and genetic risk. *CI* Confidence interval, *HR* Hazard ratio

Furthermore, we found that compared to non-laxative exposed participants, those with use of senna (HR, 1.67; 95 CI: 1.34–2.09), lactulose (HR, 1.50; 95% CI: 1.24–1.81), and docusate (HR, 2.12; 95% CI: 1.54–2.91) were positively associated with the risk of developed incident dementia (Supplementary Table 7). We further evaluated the association between the dosage of senna, lactulose, and docusate and the risk of incident dementia. Compared to non-laxative exposed participants, those with low and high dosage of senna was associated with 1.53 (95% CI: 1.11–2.11) and 1.82 (95% CI: 1.34–2.47) greater risk of dementia (Supplementary Table 8). Likewise, high dosage of lactulose (HR, 1.88; 95% CI: 1.49–2.37) and docusate (HR, 2.38; 95% CI: 1.57–3.62) was associated with increased risk of dementia.

### Joint effect of laxative use and genetic susceptibility on dementia risk

**Fig. 2:**
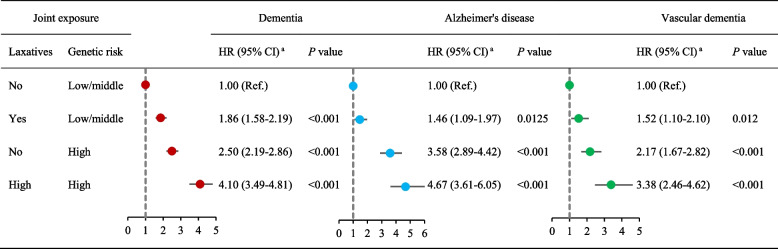
Hazards ratios (HRs) and 95% confidence interval (CIs) of dementia by joint exposures of laxatives use and genetic predisposition. ^a^ propensity-score match models were adjusted for sex, age, ethnicity, socioeconomic status, education attainment, current employment status, smoking status, alcohol consumption, physical activity, diet, BMI, heart disease, stroke, diabetes, hypertension, depression, constipation, and cholesterol levels. CI, confidence interval; HR, hazard ratio. Measures of additive interaction for dementia: Relative excess risk due to interaction (RERI): 0.736, 95% CI: 0.127, 1.346; Attributable proportion due to interaction (AP): 0.180, 95% CI: 0.047, 0.312; Synergy index (SI): 1.312, 95% CI: 1.050, 1.639. Measures of additive interaction for Alzheimer’s disease: Relative excess risk due to interaction (RERI):0.636, 95% CI: − 0.447, 1.719; Attributable proportion due to interaction (AP): 0.136, 95% CI: − 0.077, 0.348; Synergy index (SI): 1.209, 95% CI: 0.878, 1.666. Measures of additive interaction for vascular dementia: Relative excess risk due to interaction (RERI):0.683, 95% CI: − 0.340, 1.707; Attributable proportion due to interaction (AP): 0.203, 95% CI: − 0.065, 0.470; Synergy index (SI): 1.404, 95% CI: 0.843, 2.338

Figure 2 shows the association of combined laxatives use and genetic risk profiles with incidence of dementia. In joint effect analysis, compared to individuals with low/middle genetic susceptibility and non-laxatives use, the HRs (95% CIs) of dementia were 1.86 (1.58–2.19) in those with low/middle genetic susceptibility plus use of laxatives, and 4.10 (3.49–4.81) for those with high genetic susceptibility plus use of laxatives. Additionally, participants with joint exposure of high genetic susceptibility and laxatives use were associated with increased risk of Alzheimer’s disease and vascular dementia, with HR (95% CI) of 4.67 (3.61–6.05) for Alzheimer’s disease, and 3.38 (2.46–4.62) for vascular dementia, compared to those with low/middle genetic susceptibility and non-laxatives use. There was an additive interaction between laxatives use and genetic susceptibility on dementia (RERI: 0.736, 95% CI: 0.127 to 1.246; AP: 0.180, 95% CI: 0.047 to 0.312; SI: 1.312, 95% CI: 1.050 to 1.639), but not Alzheimer’s disease and vascular dementia.

### Multiplication interaction and stratification analyses

**Fig. 3:**
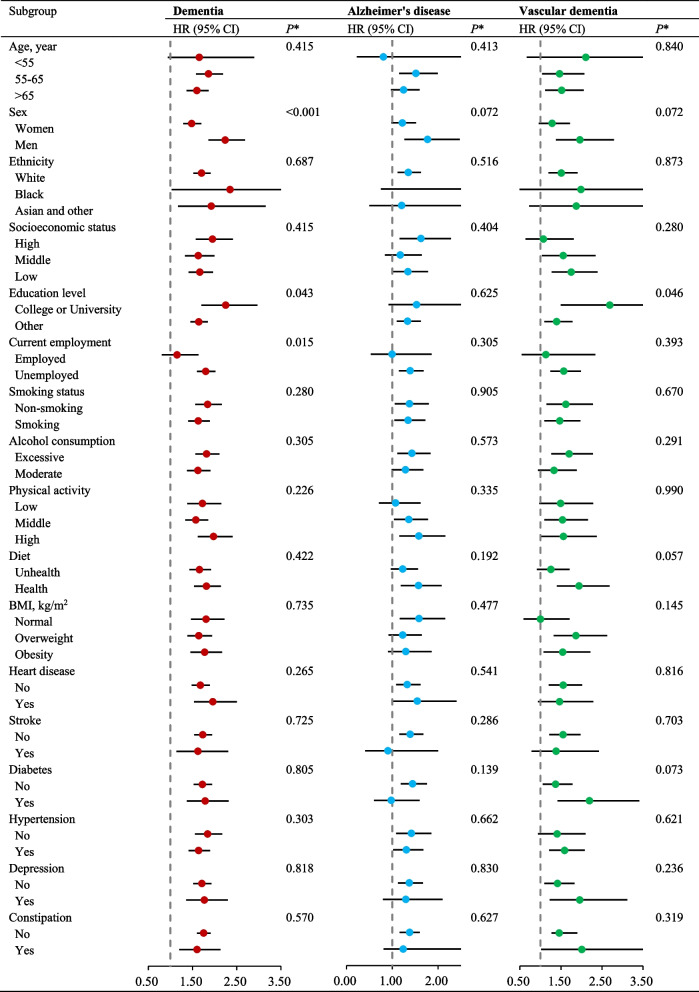
Propensity score matched (PSM) association of laxatives use with incidence of dementia according to baseline factors. *Note:* propensity-score match models were adjusted for sex, age, ethnicity, socioeconomic status, education attainment, current employment status, smoking status, alcohol consumption, physical activity, diet, BMI, heart disease, stroke, diabetes, hypertension, depression, cholesterol levels, constipation, and genetic risk. CI, confidence interval; HR, hazard ratio. *The *P* value used for heterogeneity corresponded to the Χ^2^ test statistic for the likelihood ratio test comparing models with and without interaction between laxatives use and baseline factors

Series stratification analysis were applied to evaluated whether the covariates modify the association between use of laxatives and incident dementia (Fig. 3). We found the increased risk of dementia in matched cohort with laxatives use did not meaningfully differ by age, ethnicity, socioeconomic status, smoking, alcohol consumption, physical activity, diet, BMI, heart disease, stroke, diabetes, hypertension, depression, and constipation (*P* for interaction > 0.05). However, we found a significant interaction between laxatives and sex on dementia, in which the adverse effect of laxatives was more evident among men (HR = 2.24, 95% CI, 1.86–2.69 among men versus HR = 1.48, 95% CI, 1.29–1.70 among women, *P* for interaction < 0.001). Moreover, the association between laxatives and dementia was more pronounced in high education level (*P* for interaction =0.043). Additionally, a significant interaction was found between employment status and laxatives in relation to incidence of dementia, and the risk effect of laxatives on dementia was more evident among participants who were unemployed (*P* for interaction =0.015).

### Additional analyses

The results were not much altered compared with those from initial analyses when we excluded participants who developed dementia or died within the first 2-year follow-up period (Supplementary Table 9) or excluded participants with major prior diseases at baseline (Supplementary Table 10), repeated the analyses using competing risk regression models (Supplementary Table 11). Furthermore, we found that compared to non-laxative exposed participants, those with use of laxatives was associated with higher risk of developed incident dementia when restricting to 0–5, 6–10, and 10+ years of follow-up (Supplementary Table 12).

## Discussion

In this retrospective cohort study, we found that the use of laxatives was associated with higher risk of dementia, Alzheimer’s disease, and vascular dementia. Stimulant laxatives, softeners and emollients, and osmotic laxatives were risk of dementia, but bulk forming laxatives was not associated with the outcome. A significantly additive interaction was observed between laxatives and genetic susceptibility on dementia. Compared to participants with low/middle genetic susceptibility and non-laxatives use, those with high genetic susceptibility and laxatives use was associated with 4.10-folds higher risk of dementia, of which 18% was due to their interaction. Additionally, the risk effect of laxatives on dementia was more evident among men.

The basic epidemiological characteristics were similar in constipation and dementia. The pathogeneses of the two disorders involve changes in intestinal flora and traits of the brain-gut axis [5], indicating that there may be a close relationship between the two. A large multicenter cohort study conducted by Ronald in US reported that constipation was an independent predictor for dementia and parkinsonism [8]. Laxatives are the main management of constipation and may be used to prevent constipation. However, the evidence of whether laxatives use could reduce the risk of dementia was limited in the UK, with stimulant laxatives being the most commonly used laxatives. A retrospective cohort study including 6188 patients (1547 cases versus 4641 controls, aged 50 years or older) in Taiwan found that patients who used magnesium oxide was associated with higher risk of dementia, but not associated with Alzheimer’s disease and vascular dementia [10]. However, our findings suggested that the use of laxatives was associated with higher risk of dementia. Magnesium oxide was used as saline laxatives, a kind of osmotic laxatives [31, 32], reduces migraines [33], blood pressure [34], and the risk of incident stroke (especially ischemic strokes) [35], and may relieve depressive symptoms and behaviors [36–38], thereby may reduce the aforementioned disorders-related dementia risk. But the major kind of osmotic laxatives in our study was lactulose product, which may cause the inconsistent results.

Our results demonstrated that the association of laxatives with dementia and its subtypes seemed to be stronger for non-Alzheimer’s disease and non-vascular dementias. The common form of dementia including Alzheimer’s disease, vascular dementia, dementia with Lewy bodies, Parkinson’s disease dementia, and frontotemporal dementia [39–41]. This study found that laxative use was associated with 1.72-folds higher risk of dementia, 1.36-folds higher risk Alzheimer’s disease, 1.53-folds higher risk of vascular dementia, and 2.15-folds higher risk of other dementia subtypes. Previous studies have reported that constipation was significantly associated with dementia with Lewy bodies and Parkinson’s disease dementia [9, 42]. Furthermore, Santos et al., reported that constipation is associated with cognitive decline in Parkinson’s disease patients but not in controls [43]. The association of laxatives with Parkinson’s disease dementia may stronger than they are with Alzheimer’s disease and vascular dementias. Furthermore, the validity of the dementia subtype diagnoses is not high, particularly for vascular dementia [44], which may influence our risk estimates. Wilkinson et al., have estimated the positive predictive value (PPV) of using UK routinely-collected healthcare datasets to identify cases of all-cause dementia, Alzheimer’s disease, and vascular dementia during follow-up in the UK Biobank, and found that PPVs for all datasets combined were 82.5% (74.5–88.8) for all-cause dementia, 71.4% (58.7–82.1) for Alzheimer’s disease, and 43.8% (19.8–70.1) for vascular dementia [44]. The lower PPVs for Alzheimer’s disease and vascular dementia may influence our risk estimates. Further prospective cohort studies are needed to verify our findings.

Our findings suggested that softeners and emollients, and osmotic laxative, and stimulant laxatives users had increased risk of dementia, but those took bulk forming laxatives was not link to dementia. The association between laxative types and dementia remains largely unknown. Previous studies found that the stepwise approach to laxative therapy was recommended begin with bulk forming laxatives—initial management for mild constipation, then an osmotic laxative followed by a stimulant laxative—management of mild to moderate constipation [45]. According to previous research, the recommended stage process strategy to laxative therapy should start with bulk forming laxatives as the initial treatment for mild constipation, followed by an osmotic laxative and a stimulant laxative as the management of mild to moderate constipation. Therefore, the relationship between the types of laxatives and dementia may reflect the link between the severity of constipation and new-onset dementia. The results also suggested that laxatives may relieve the constipation symptoms but fail to reduce the risk of dementia caused by the pathological mechanism of constipation.

The importance of genetic basis on the development dementia has been revealed previously. In the current study, it has been possible to quantify the genetic risk for dementia using PRS combining numerous risk alleles. According to our findings, a high genetic risk score was linked to a roughly 2.8-fold greater risk of dementia than a low genetic risk score. Furthermore, genetic susceptibility and laxative use showed a strong additive interaction, and individuals with high genetic susceptibility plus use of laxatives was associated with more than 4-fold higher risk of incident dementia compared to those with low/middle genetic susceptibility plus non-laxatives use. The findings suggested that attention should be paid to the relationship between laxatives use and dementia, especially in people at high genetic susceptibility.

Further, our study suggested a significant interaction between laxatives and sex on dementia, in which the risk effect of laxatives was more evident among men (men: HR, 2.24; women: HR, 1.48), suggesting that men are particularly susceptible to the adverse effects of constipation on developing dementia. Although the specific explain of these sex differences is unknown, it seems certain that a variety of biological and psychological mechanisms have a role, such as hormonal, psychological, and social factors.

Our research revealed a significantly additive interaction between laxatives use and genetic susceptibility on dementia. There are several mechanisms whereby the joint association of laxatives and genetic susceptibility may be related to dementia. Athanasios et al. conducted a case-control study to identify the protein expression alterations in irritable bowel syndrome (IBS) patients compared to healthy individuals and found that IBS-constipation group overexpressed apolipoprotein H (APOH) [46]. A multifunctional plasma glycoprotein known as APOH has been linked to adverse health effects [47], and containing in several physiological processes including lipid metabolism, apoptosis, inflammation, and atherogenesis [48]. Additionally, APOH levels have been associated to Alzheimer’s disease, moderate cognitive impairment, the predementia syndrome, and cognitive aging [49]. Therefore, constipation and genetic susceptibility may perform joint role in the pathogenesis of dementia. Unhealthy lifestyle, such as unhealthy diet (e.g., low fiber, high protein, or low intake) and sedentary lifestyle, low socio-economic status, hyperglycemia, depression, diabetes, and stroke were common causes of constipation and conditions associated with constipation [50–52]. There were interactions between dietary protein/physical inactive/low socio-economic status/depression/diabetes/stroke and dementia related genetic factors [53–59], and these conditions may play a joint effect in the pathogenesis of dementia. Additionally, the interaction between laxatives and genetic risk for dementia may have arisen due to chance. Exploring the potential joint effects of laxatives and genes on dementia requires more validation in animal studies.

Our study’s advantages included the large sample size, the long-term follow-up duration, the standardized data collection protocol, and the use of propensity score matching design that effectively balanced a wide range of the confounding factors. There are also limitations in this study. First, the assessment of medication from the questionnaire and medical record may not reflect actual drug exposure, which may influence our risk estimates. Second, although most laxative types are collected, but several laxative types may still not be recorded, such as magnesium oxide. Third, we diagnosed dementia using self-reported, medical records, and death registration. The patients with early or mild dementia may not be documented in the hospital records, leading to an underestimation of the prevalence of dementia. Fourth, although we conducted analysis using propensity matching, inverse probability weighting, and fully adjusted Cox regression to assess the validity of the study, the bias remains for unhealthy individuals at particular risk of developing further conditions, such as dementia, are treated with more medications. Fifth, the validity of the dementia subtype diagnoses is not high, particularly for vascular dementia [44], which may influence our risk estimates. Furthermore, although all analyses in the present study were adjusted for known potential sources of bias, the possibility of unmeasured confounding factors and reverse causation remains. Additionally, information on laxatives was assessment only once at baseline and the time of treatment initiation was not recorded, which may limit our data to assess time-varying hazards and drug effects associated with treatment duration. Population-based prospective cohort studies using new-user active comparator design are needed to investigate the association of laxatives and dementia. Although we conducted analysis using propensity matching, inverse probability weighting, and fully adjusted Cox regression to assess the validity of the study, the bias remains for unhealthy individuals at particular risk of developing further conditions, such as dementia, are treated with more medications. Finally, the majority of the participants in the current study were white British, limiting generalization our findings to other ethnic groups.

Despite advances in understanding the pathophysiology of dementia, clinical treatment of dementia continues to be suboptimal. Therefore, identifying risk factors for dementia is of high priority. Our findings raise the possibility of a potentially positive association between laxative use and the risk of dementia, although causality cannot be inferred. The finding suggested that the use of laxatives could not reduce the risk of dementia through treating constipation. Constipation is one of the most common gastrointestinal disorders, and more than one third of people over 65 years are affected by constipation during their lifetime. From a public health perspective, even small potential risks associated with constipation may have important public health implications. Therefore, attention should be paid to early prevention of constipation or to find effective ways to prevent dementia that related to constipation.

## Conclusions

In summary, use of laxatives, including softeners and emollients, stimulant laxatives, and osmotic laxatives, was positively associated with the risk of dementia. Moreover, the risk effect of laxatives on dementia was more evident among men. Joint exposures of laxatives and genetic predisposition was associated with higher risk of dementia than either condition alone. A significantly additive interaction was observed between laxatives and genetic susceptibility on dementia. Our findings suggested that attention should be paid to the relationship between laxatives use and dementia, especially in people at high genetic susceptibility.

## Supplementary Information

**Additional file 1: Supplementary Methods.** Polygenic risk score (PRS). **Supplementary Table 1.** Codes used in the UK Biobank study to identify dementia cases. **Supplementary Table 2.** Information on laxative types. **Supplementary Table 3.** Single nucleotide polymorphisms (SNPs) applied to generate the polygenetic risk score for dementia. **Supplementary Table 4.** Proportional hazards assumption test. **Supplementary Table 5.** Association between genetic susceptibility and the risk of dementia. **Supplementary Table 6.** Incidence rates per 1000 person-years of dementia for laxative use and non-use groups. **Supplementary Table 7.** Hazards ratios (HRs) and 95% confidence interval (CIs) of dementia by joint exposures of laxatives use and genetic predisposition after excluding first 2 years incidence of dementia or death during follow-up. **Supplementary Table 8.** Hazards ratios (HRs) and 95% confidence interval (CIs) of dementia by joint exposures of laxatives use and genetic predisposition after excluding participants with major prior diseases (e.g., heart disease, stroke, diabetes, and cancer) at baseline. **Supplementary Table 9.** Association of laxatives use with incidence of dementia: results from competing risk models. **Supplementary Table 10.** Association of laxatives use with incidence of dementia when restricting to 0–5, 6–10, and 10+ years of follow-up. **Supplementary Table 11.** Association of detail laxative types with incidence of dementia. **Supplementary Table 12.** Association of laxative dosage with incidence of dementia. **Supplementary Fig. 1.** Standardized mean differences (SMD) before and after matching. **Supplementary Fig. 2.** The risk of incidence of dementia according to genetic risk.

## Data Availability

The data that support the findings of this study are available from UK Biobank (https://www.ukbiobank.ac.uk/), but restrictions apply to the availability of these data, which were used under license for the current study, and so are not publicly available. Data are however available from the authors upon reasonable request and with permission of UK Biobank.
