# Supplementary material for: Association of laxatives use with incident dementia and modifying effect of genetic susceptibility: a population-based cohort study with propensity score matching
Source: BMC Geriatr. 2023 Mar 4;23:122. doi: 10.1186/s12877-023-03854-w (PMC9985868; doi:10.1186/s12877-023-03854-w)
Supplement: Supplementary file 1 — Additional file 1: Supplementary Methods. Polygenic risk score (PRS). Supplementary Table 1. Codes used in the UK Biobank study to identify dementia cases. Supplementary Table 2. Information on laxative types. Supplementary Table 3. Single nucleotide polymorphisms (SNPs) applied to generate the polygenetic risk score for dementia. Supplementary Table 4. Proportional hazards assumption test. Supplementary Table 5. Association between genetic susceptibility and the risk of dementia. Supplementary Table 6. Incidence rates per 1000 person-years of dementia for laxative use and non-use groups. Supplementary Table 7. Hazards ratios (HRs) and 95% confidence interval (CIs) of dementia by joint exposures of laxatives use and genetic predisposition after excluding first 2 years incidence of dementia or death during follow-up. Supplementary Table 8. Hazards ratios (HRs) and 95% confidence interval (CIs) of dementia by joint exposures of laxatives use and genetic predisposition after excluding participants with major prior diseases (e.g., heart disease, stroke, diabetes, and cancer) at baseline. Supplementary Table 9. Association of laxatives use with incidence of dementia: results from competing risk models. Supplementary Table 10. Association of laxatives use with incidence of dementia when restricting to 0–5, 6–10, and 10+ years of follow-up. Supplementary Table 11. Association of detail laxative types with incidence of dementia. Supplementary Table 12. Association of laxative dosage with incidence of dementia. Supplementary Fig. 1. Standardized mean differences (SMD) before and after matching. Supplementary Fig. 2. The risk of incidence of dementia according to genetic risk. [file 12877_2023_3854_MOESM1_ESM.docx]

**Supplementary materials**

**Supplementary Methods:** Polygenic risk score (PRS)

**Supplementary Table 1.** Codes used in the UK Biobank study to identify dementia cases

**Supplementary Table 2.** Information on laxative types

**Supplementary Table 3.** Single nucleotide polymorphisms (SNPs) applied to generate the polygenetic risk score for dementia

**Supplementary Table 4.** Proportional hazards assumption test

**Supplementary Table 5.** Association between genetic susceptibility and the risk of dementia

**Supplementary Table 6.** Incidence rates per 1000 person-years of dementia for laxative use and non-use groups

**Supplementary Table 7.** Hazards ratios (HRs) and 95% confidence interval (CIs) of dementia by joint exposures of laxatives use and genetic predisposition after excluding first 2 years incidence of dementia or death during follow-up

**Supplementary Table 8.** Hazards ratios (HRs) and 95% confidence interval (CIs) of dementia by joint exposures of laxatives use and genetic predisposition after excluding participants with major prior diseases (e.g., heart disease, stroke, diabetes, and cancer) at baseline

**Supplementary Table 9.** Association of laxatives use with incidence of dementia: results from competing risk models

**Supplementary Table 10.** Association of laxatives use with incidence of dementia when restricting to 0-5, 6-10, and 10+ years of follow-up

**Supplementary Table 11.** Association of detail laxative types with incidence of dementia

**Supplementary Table 12.** Association of laxative dosage with incidence of dementia

**Supplementary Figure 1.** Standardized mean differences (SMD) before and after matching

**Supplementary Figure 2**. The risk of incidence of dementia according to genetic risk

**Methods:** **Polygenic risk score (PRS)**

To generate a polygenic risk score (PRS), each single nucleotide polymorphism (SNP) was recoded as 0, 1, or 2 according to the number of risk-increasing alleles. The score was calculated using the equation:

Score=β_1_×SNP_1_+β_2_×SNP_2_+…+β_n-1_×SNP_n-1_ +…+β_n_×SNP_n_

where n is the total number of SNPs, and β is per-allele log odds ratio (OR) for associated with SNP_n_. The effect size estimates of β were taken from a genome wide association study that carried out in subjects from Global Urate Genetics Consortium study of individuals of European ancestry. PRS were categorized as low, middle, and high according to tertiles of polygenic risk score.

**Supplementary Table 1. Codes used in the UK Biobank study to identify dementia cases**

| **Dementia** | **ICD codes** |
| --- | --- |
| All-cause dementia | ICD-9 codes 290.2, 290.3, 290.4, 291.2, 294.1, 331.0, 331.1, 331.2, 331.5;  ICD-10 codes A81.0, F00.0, F00.1, F00.9, F01.0, F01.1, F01.2, F01.3, F01.8, F01.9, F02.0, F02.1, F02.2, F02.3, F02.4, F02.8, F03, F05.1, F10.6, G30.0, G30.1, G30.8, G30.9, G31.0, G31.1, G31.8, I67.3 |
| Alzheimer’s disease | ICD-9 codes 331.0;  ICD-10 codes F00.0, F00.1, F00.2, F00.9, G30.0, G30.1, G30.8, G30.9 |
| Vascular dementia | ICD-9 codes 290.4;  ICD-10 codes F01.0, F01.1, F01.2, F01.3, F01.8, F01.9, I67.3. |
| Other types of dementia (such as frontotemporal dementia, dementia with Lewy bodies) | ICD-9 codes 290.2, 290.3, 291.2, 294.1, 331.1, 331.2, 331.5;  ICD-10 codes A81.0, F02, F03, F05.1, F10.6, G31.0, G31.1, G31.8. |

*Abbreviations: ICD* International Classification of Diseases

**Supplementary Table 2. Information on laxative types**

| **Bulk Forming Laxatives** | |
| --- | --- |
| 1140865752 | Ispaghula husk |
| 1140865778 | Sterculia |
| 1140865780 | Sterculia+frangula 62%/8% granules |
| 1140865772 | Methylcellulose |
| 1140865774 | Celevac 500mg tablet |
| 1140865408 | Mebeverine hcl+ispaghula 135mg/3.5g/sachet granules |
| 1141157494 | Ispaghula husk |
| 1140865762 | Regulan 3.6g/sachet powder |
| **Softeners and Emollients** | |
| 1140879404 | Docusate sodium |
| 1140881530 | Liquid paraffin+magnesium hydroxide oral emulsion bp |
| 1140916270 | Liquid paraffin+white soft paraffin 50%/50% ointment |
| 1141170844 | Liquid paraffin product |
| 1140865734 | Liquid paraffin |
| 1140865792 | Petrolagar emulsion |
| **Osmotic Laxatives** | |
| 1140865800 | Lactulose product |
| 1140865878 | Lactitol |
| 1140925794 | Polyethylene glycol product |
| 1140864408 | Fleet enema |
| 1140865840 | Predfoam 20mg enema |
| 1140865896 | Fletchers' arachis oil retention enema |
| 1140865906 | Arachis oil retention enema |
| 1140865910 | Magnesium sulphate 50% retention enema |
| 1140865694 | Danthron |
| 1140865702 | Normax capsule |
| 1140865726 | Picolax 10mg/sachet oral powder |
| 1140865804 | Duphalac solution |
| 1140865808 | Lactugal solution |
| 1140865810 | Regulose solution |
| 1140865876 | Citramag 17.7g/sachet powder |
| 1140881434 | Fletchers' magnesium sulphate 50% retention enema |
| 1140881436 | Fletchers' phosphate standard tube enema |
| 1140881514 | Fletchers' micro-enema |
| 1140881516 | Norgalax 120mg micro-enema |
| 1140883074 | Micolette micro-enema |
| 1140883076 | Micralax micro-enema |
| 1140883078 | Relaxit micro-enema |
| **Stimulant Laxatives** | |
| 1140865686 | Dulco-lax 5mg e/c tablet |
| 1140865786 | Bisacodyl |
| 1140865716 | Senna |
| 1141157178 | Senna product |
| 1141191184 | Nylax with senna 7.5mg tablet |
| 1140865724 | Sodium picosulphate |
| 1140909704 | Sodium picosulfate |
| 1140851088 | Senokot 7.5mg tablet |
| 1141200498 | Senokot hi-fibre lemon 3.5g/sachet s/f granules |

**Supplementary Table 3. Single nucleotide polymorphisms (SNPs) applied to generate the polygenetic risk score for dementia**

| CHR | Position | SNP | Closed Genes | A1 | A2 | Effect size |
| --- | --- | --- | --- | --- | --- | --- |
| 1 | 207802552 | rs4844610 | CR1 | A | A | 0.16 |
| 2 | 127892810 | rs6733839 | BIN1 | T | T | 0.18 |
| 2 | 233981912 | rs10933431 | INPPSD | C | G | 0.09 |
| 5 | 88223420 | rs190982 | MEF2C | A | G | 0.06 |
| 6 | 41129252 | rs75932628 | TREM2 | T | T | 0.73 |
| 6 | 47431284 | rs9473117 | CD2AP | C | C | 0.09 |
| 6 | 41129207 | rs143332484 | TREM2 | T | T | 0.46 |
| 7 | 100091795 | rs12539172 | ZCWPW1 | T | T | 0.08 |
| 7 | 143099133 | rs10808026 | EPHA1 | A | A | 0.11 |
| 7 | 37844263 | rs4723711 | NME8 | A | T | 0.06 |
| 8 | 27219987 | rs73223431 | PTK2B | T | T | 0.10 |
| 8 | 27467686 | rs9331896 | CLU | T | C | 0.13 |
| 11 | 47380340 | rs3740688 | CELF1 | T | G | 0.08 |
| 11 | 59936926 | rs7933202 | MS4A2 | A | C | 0.12 |
| 11 | 85868640 | rs3851179 | PICALM | C | T | 0.13 |
| 11 | 121435587 | rs11218343 | SORLI | T | C | 0.22 |
| 14 | 53391680 | rs17125924 | FERMT2 | G | G | 0.13 |
| 14 | 92932828 | rs12881735 | SLC24A4 | T | C | 0.08 |
| 15 | 59045774 | rs593742 | ADAM10 | A | G | 0.07 |
| 16 | 19808163 | rs7185636 | IQCK | T | C | 0.08 |
| 16 | 79355857 | rs62039712 | wWOX | A | A | 0.15 |
| 16 | 81942028 | rs72824905 | PLCG2 | C | C | 0.43 |
| 17 | 61538148 | rs138190086 | ACE | A | A | 0.26 |
| 17 | 47297297 | rs616338 | ABI3 | T | T | 0.35 |
| 19 | 45411941 | rs429358 | APOE | C | T | 0.99 |
| 19 | 45412079 | rs7412 | APOE | T | C | 0.65 |
| 19 | 1056492 | rs3752246 | ABCA7 | G | G | 0.14 |
| 20 | 54997568 | rs6024870 | CASS4 | G | A | 0.13 |
| 21 | 28156856 | rs2830500 | ADAMTSI | C | A | 0.07 |

**Supplementary Table 4. Proportional hazards assumption test**

| Factors | rho | χ^2^ | *P* value |
| --- | --- | --- | --- |
| Sex | -0.05047 | 3.77 | 0.0521 |
| Age | 0.04823 | 3.19 | 0.0742 |
| Ethnicity | -0.03396 | 1.6 | 0.2059 |
| Socioeconomic status | -0.03431 | 1.65 | 0.1986 |
| Education level | 0.03922 | 2.11 | 0.1465 |
| Current employment | 0.02039 | 0.62 | 0.4301 |
| Smoking status | 0.02917 | 1.19 | 0.2752 |
| Alcohol consumption | 0.0289 | 1.14 | 0.2848 |
| Physical activity | 0.02629 | 0.98 | 0.3218 |
| Diet | 0.00386 | 0.02 | 0.8831 |
| BMI | 0.03385 | 1.69 | 0.1936 |
| Heart disease | -0.03864 | 2.15 | 0.1428 |
| Stroke | -0.00317 | 0.01 | 0.9045 |
| Hypertension | 0.00318 | 0.01 | 0.9057 |
| Diabetes | 0.00513 | 0.04 | 0.8485 |
| Depression | -0.03305 | 1.5 | 0.2202 |
| Cholesterol | 0.00379 | 0.02 | 0.8855 |
| Constipation | -0.03583 | 1.77 | 0.1832 |
| Genetic risk | 0.03147 | 1.36 | 0.2431 |
| Global test |  | 28.25 | 0.0787 |

**Supplementary Table 5.** **Association between genetic susceptibility and the risk of dementia**

| Genetic risk categories | Dementia  HR (95% CI) |  | Alzheimer’s disease  HR (95% CI) |  | Vascular dementia  HR (95% CI) |
| --- | --- | --- | --- | --- | --- |
| Low | 1.00 (ref.) |  | 1.00 (ref.) |  | 1.00 (ref.) |
| Middle | 1.29 (1.21-1.38) |  | 1.42 (1.26-1.59) |  | 1.24 (1.08-1.42) |
| High | 2.76 (2.60-2.93) |  | 4.11 (3.71-4.54) |  | 2.43 (2.15-2.75) |

*Note:* Cox regression models were adjusted for sex, age, ethnicity, socioeconomic status, education attainment, current employment status, smoking status, alcohol consumption, physical activity, diet, BMI, heart disease, stroke, diabetes, hypertension, depression, cholesterol levels, and constipation. CI, confidence interval; HR, hazard ratio.

**Supplementary Table 6. Incidence rates per 1000 person-years of dementia for laxative use and non-use groups**

|  | No. of cases | Person-years | Incidence rates  (per 1000 person-years) |
| --- | --- | --- | --- |
| Dementia | 1377 | 730899 | 1.88 |
| Non-Laxatives use | 877 | 548866 | 1.60 |
| Laxatives use | 500 | 182032 | 2.75 |
| Alzheimer's disease | 539 | 734508 | 0.73 |
| Non-Laxatives use | 371 | 550985 | 0.67 |
| Laxatives use | 168 | 183522 | 0.92 |
| Vascular dementia | 343 | 735157 | 0.47 |
| Non-Laxatives use | 227 | 551476 | 0.41 |
| Laxatives use | 116 | 183681 | 0.63 |

**Supplementary Table 7. Association of detail laxative types with incidence of dementia**

|  | Non-laxatives  users | Dementia | | Alzheimer's disease | | Vascular dementia | |
| --- | --- | --- | --- | --- | --- | --- | --- |
|  |  | HR (95% CI) ^a^ | *P*  value | HR (95% CI) | *P*  value | HR (95% CI) ^a^ | *P*  value |
| Senna | 1.00 (ref.) | 1.67 (1.34-2.09) | <0.001 | 1.72 (1.21-2.43) | 0.002 | 2.21 (1.52-3.24) | <0.001 |
| Lactulose | 1.00 (ref.) | 1.50 (1.25-1.81) | <0.001 | 1.52 (1.14-2.01) | 0.004 | 1.79 (1.27-2.53) | 0.001 |
| Docusate | 1.00 (ref.) | 2.12 (1.54-2.91) | <0.001 | 1.73 (1.00-2.99) | 0.049 | 2.37 (1.30-4.29) | 0.005 |

^a^ Models were adjusted for sex, age, ethnicity, socioeconomic status, education attainment, current employment status, smoking status, alcohol consumption, physical activity, diet, BMI, heart disease, stroke, diabetes, hypertension, depression, cholesterol levels, constipation, and genetic risk. CI, confidence interval; HR, hazard ratio.

**Supplementary Table 8. Association of laxative dosage with incidence of dementia**

| Dosage | Dementia | | Alzheimer's disease | | Vascular dementia | |
| --- | --- | --- | --- | --- | --- | --- |
|  | HR (95% CI) ^a^ | *P*  value | HR (95% CI) | *P*  value | HR (95% CI) ^a^ | *P*  value |
| **Senna** |  |  |  |  |  |  |
| Non-laxative use | 1.00 (ref.) |  | 1.00 (ref.) |  | 1.00 (ref.) |  |
| Low | 1.53 (1.11-2.12) | 0.010 | 1.60 (0.98-2.62) | 0.062 | 2.26 (1.33-3.84) | 0.003 |
| High | 1.82 (1.34-2.47) | <0.001 | 1.84 (1.14-2.98) | 0.013 | 2.16 (1.27-3.67) | 0.005 |
| **Lactulose** |  |  |  |  |  |  |
| Non-laxative use | 1.00 (ref.) |  | 1.00 (ref.) |  | 1.00 (ref.) |  |
| Low | 1.12 (0.83-1.51) | 0.472 | 1.11 (0.70-1.77) | 0.661 | 0.77 (0.36-1.62) | 0.485 |
| High | 1.88 (1.49-2.38) | <0.001 | 1.92 (1.34-2.74) | <0.001 | 2.71 (1.84-3.99) | <0.001 |
| **Docusate** |  |  |  |  |  |  |
| Non-laxative use | 1.00 (ref.) |  | 1.00 (ref.) |  | 1.00 (ref.) |  |
| Low | 1.82 (1.11-2.98) | 0.017 | 1.08 (0.41-2.89) | 0.874 | 1.96 (0.81-4.73) | 0.134 |
| High | 2.38 (1.57-3.62) | <0.001 | 2.34 (1.21-4.51) | 0.011 | 2.84 (1.27-6.35) | 0.011 |

^a^ Models were adjusted for sex, age, ethnicity, socioeconomic status, education attainment, current employment status, smoking status, alcohol consumption, physical activity, diet, BMI, heart disease, stroke, diabetes, hypertension, depression, cholesterol levels, constipation, and genetic risk. CI, confidence interval; HR, hazard ratio.

**Supplementary Table 9. Hazards ratios (HRs) and 95% confidence interval (CIs) of dementia by joint exposures of laxatives use and genetic predisposition after excluding first 2 years incidence of dementia or death during follow-up**

| Joint exposure | |  | Dementia | | Alzheimer's disease | | Vascular dementia | |
| --- | --- | --- | --- | --- | --- | --- | --- | --- |
| Laxatives | Genetic risk |  | HR (95% CI) | *P* value | HR (95% CI) | *P* value | HR (95% CI) | *P* value |
| No | Low/middle |  | 1.00 (Ref.) |  | 1.00 (Ref.) |  | 1.00 (Ref.) |  |
| Yes | Low/middle |  | 1.84 (1.56-2.17) | <0.001 | 1.43 (1.06-1.93) | 0.0205 | 1.52 (1.10-2.11) | 0.012 |
| No | High |  | 2.51 (2.19-2.88) | <0.001 | 3.53 (2.85-4.37) | <0.001 | 2.16 (1.66-2.81) | <0.001 |
| High | High |  | 4.09 (3.48-4.81) | <0.001 | 4.62 (3.56-6.00) | <0.001 | 3.27 (2.37-4.51) | <0.001 |

*Note:* propensity-score match models were adjusted for sex, age, ethnicity, socioeconomic status, education attainment, current employment status, smoking status, alcohol consumption, physical activity, diet, BMI, heart disease, stroke, diabetes, hypertension, depression, cholesterol levels, and constipation. CI, confidence interval; HR, hazard ratio.

| **Measures of additive interaction for dementia:** | |
| --- | --- |
| Relative excess risk due to interaction (RERI): 0.740, 95% CI: 0.124, 1.356 | |
| Attributable proportion due to interaction (AP): 0.181, 95% CI: 0.047, 0.315 | |
| Synergy index (SI): 1.315, 95% CI: 1.049, 1.649. | |
|  |  |
| **Measures of additive interaction for Alzheimer's disease:** | |
| Relative excess risk due to interaction (RERI):0.666, 95% CI: -0.414, 1.746 | |
| Attributable proportion due to interaction (AP): 0.144, 95% CI: -0.069, 0.357 | |
| Synergy index (SI): 1.225, 95% CI: 0.885, 1.698 | |
|  |  |
| **Measures of additive interaction for vascular dementia:** | |
| Relative excess risk due to interaction (RERI):0.593, 95% CI: -0.429, 1.616 | |
| Attributable proportion due to interaction (AP): 0.181, 95% CI: -0.099, 0.461 | |
| Synergy index (SI): 1.354, 95% CI: 0.802, 2.284 | |

**Supplementary Table 10. Hazards ratios (HRs) and 95% confidence interval (CIs) of dementia by joint exposures of laxatives use and genetic predisposition** **after excluding participants with major prior diseases (e.g., heart disease, stroke, diabetes, and cancer) at baseline**

| Joint exposure | |  | Dementia | | Alzheimer's disease | | Vascular dementia | |
| --- | --- | --- | --- | --- | --- | --- | --- | --- |
| Laxatives | Genetic risk |  | HR (95% CI) | *P* value | HR (95% CI) | *P* value | HR (95% CI) | *P* value |
| No | Low/middle |  | 1.00 (Ref.) |  | 1.00 (Ref.) |  | 1.00 (Ref.) |  |
| Yes | Low/middle |  | 1.76 (1.43-2.17) | <0.001 | 1.54 (1.06-2.22) | 0.022 | 1.35 (0.87-2.11) | 0.184 |
| No | High |  | 2.73 (2.31-3.22) | <0.001 | 4.14 (3.18-5.37) | <0.001 | 1.96 (1.38-2.79) | <0.001 |
| High | High |  | 4.45 (3.65-5.41) | <0.001 | 5.76 (4.24-7.84) | <0.001 | 2.80 (180-4.35) | <0.001 |

*Note:* propensity-score match models were adjusted for sex, age, ethnicity, socioeconomic status, education attainment, current employment status, smoking status, alcohol consumption, physical activity, diet, BMI, hypertension, depression, constipation, and cholesterol levels. CI, confidence interval; HR, hazard ratio.

| **Measures of additive interaction for dementia:** | |
| --- | --- |
| Relative excess risk due to interaction (RERI): 0.957, 95% CI: 0.167, 1.747 | |
| Attributable proportion due to interaction (AP): 0.215, 95% CI: 0.062, 0.369 | |
| Synergy index (SI): 1.384, 95% CI: 1.060, 1.803. | |
|  |  |
| **Measures of additive interaction for Alzheimer's disease:** | |
| Relative excess risk due to interaction (RERI):1.090, 95% CI: -0.401, 2.582 | |
| Attributable proportion due to interaction (AP): 0.189, 95% CI: -0.037, 0.416 | |
| Synergy index (SI): 1.297, 95% CI: 0.916, 1.837 | |
|  |  |
| **Measures of additive interaction for vascular dementia:** | |
| Relative excess risk due to interaction (RERI):0.484, 95% CI: -0.764, 1.731 | |
| Attributable proportion due to interaction (AP): 0.173, 95% CI: -0.230, 0.576 | |
| Synergy index (SI): 1.368, 95% CI: 0.604, 3.098 | |

**Supplementary Table 11. Association of laxatives use with incidence of dementia: results from competing risk models**

|  | Dementia | Alzheimer's disease | Vascular dementia |
| --- | --- | --- | --- |
| No. of cases | 1377 | 539 | 343 |
| No. of death | 4761 | 5189 | 5246 |
| HR (95% CI) | 1.75 (1.57-1.96) | 1.39 (1.16-1.67) | 1.57 (1.26-1.97) |
| *P* value | <0.001 | <0.001 | <0.001 |

*Note:* Cox regression models were adjusted for sex, age, ethnicity, socioeconomic status, education attainment, current employment status, smoking status, alcohol consumption, physical activity, diet, BMI, heart disease, stroke, diabetes, hypertension, depression, cholesterol levels, constipation, and genetic risk. CI, confidence interval; HR, hazard ratio.

**Supplementary Table 12. Association of laxatives use with incidence of dementia when restricting to 0-5, 6-10, and 10+ years of follow-up**

|  | No. of new incident events | HR (95% CI) | *P* value |
| --- | --- | --- | --- |
| 0-5 years of follow-up | 176 | 1.87 (1.38-2.57) | <0.001 |
| 6-10 years of follow-up | 613 | 1.85 (1.57-2.17) | <0.001 |
| 10+ years of follow-up | 588 | 1.50 (1.26-1.77) | <0.001 |

*Note:* Cox regression models were adjusted for sex, age, ethnicity, socioeconomic status, education attainment, current employment status, smoking status, alcohol consumption, physical activity, diet, BMI, heart disease, stroke, diabetes, hypertension, depression, cholesterol levels, constipation, and genetic risk. CI, confidence interval; HR, hazard ratio.

**
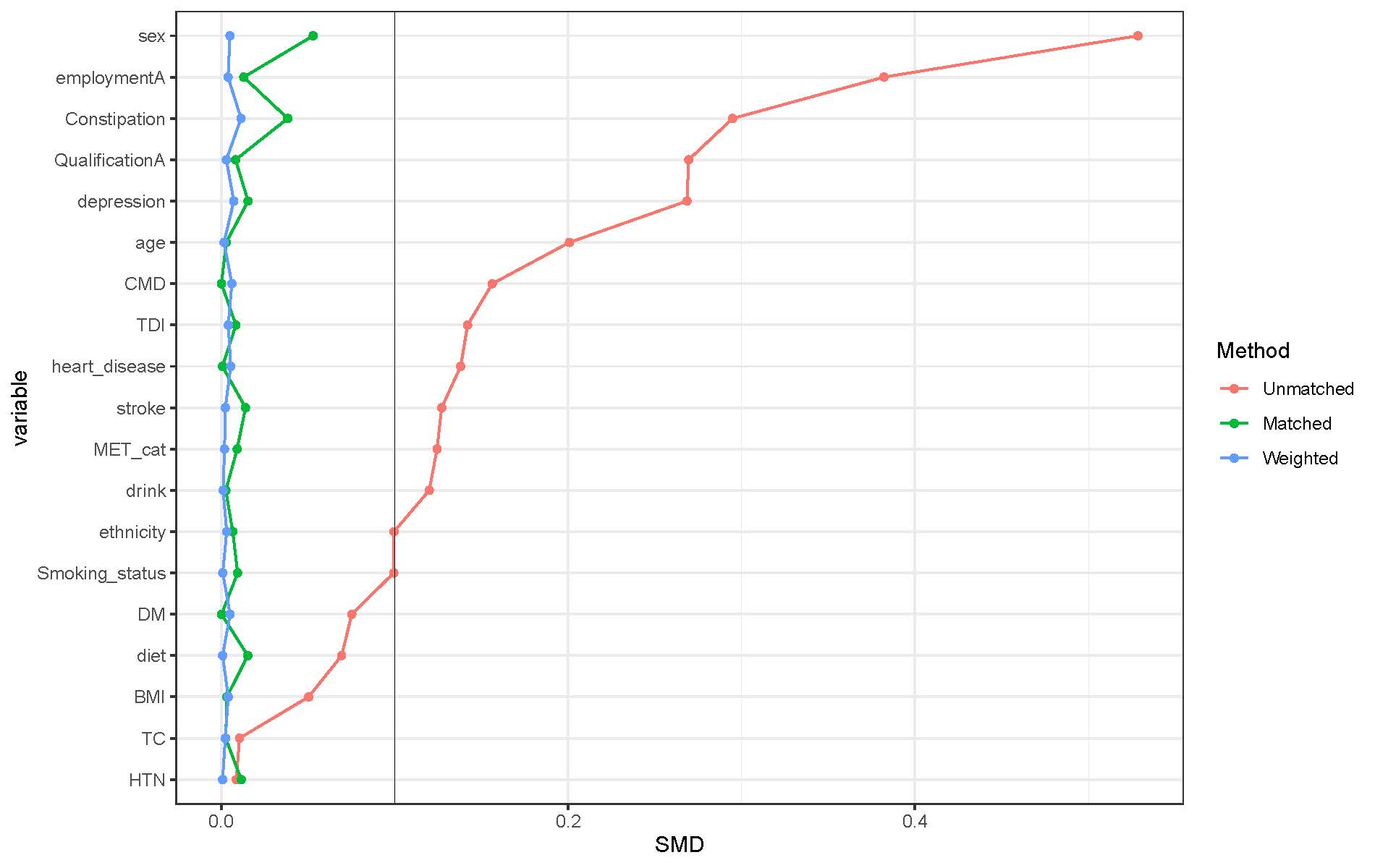
**

**Supplementary Figure 1.** Standardized mean differences (SMD) before and after matching


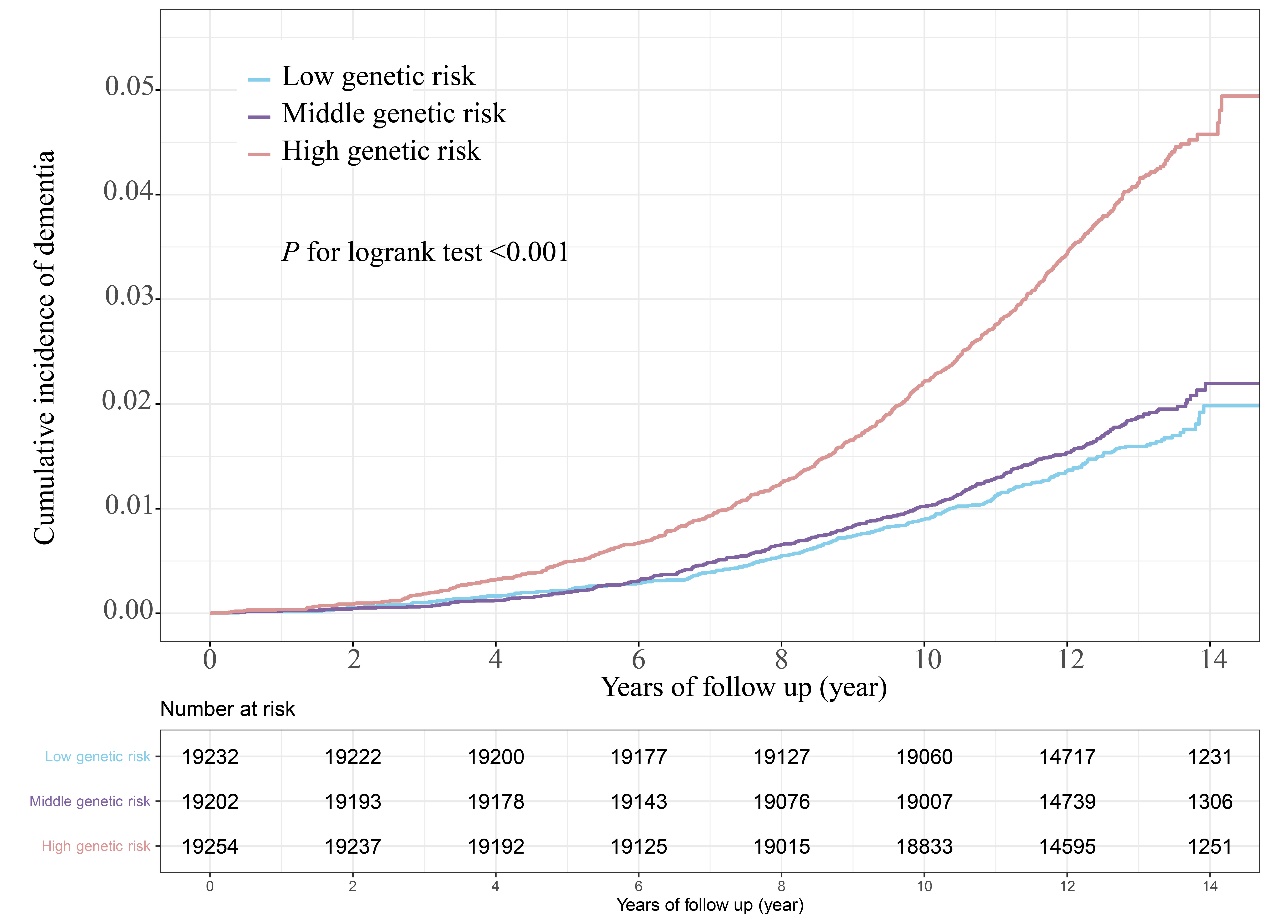


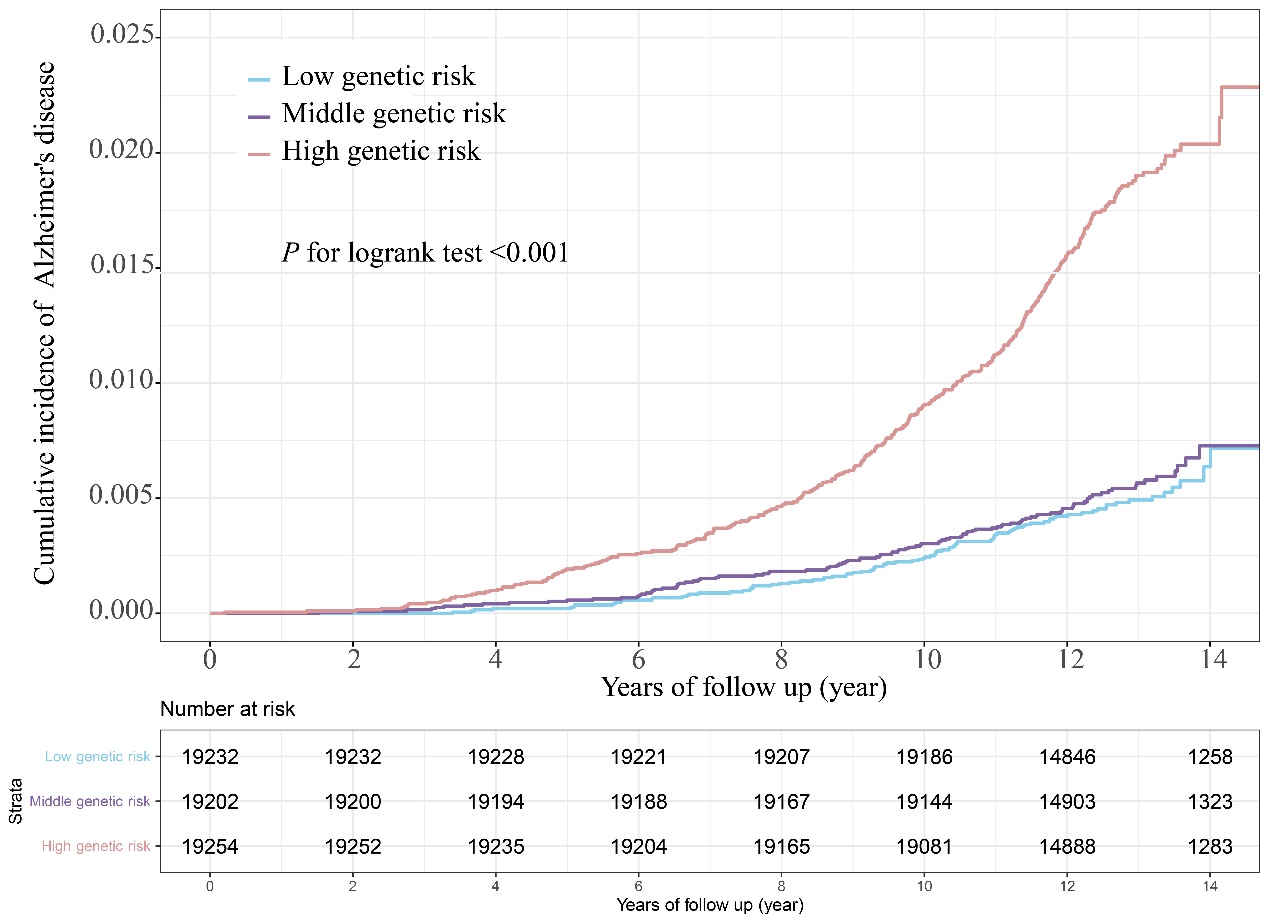


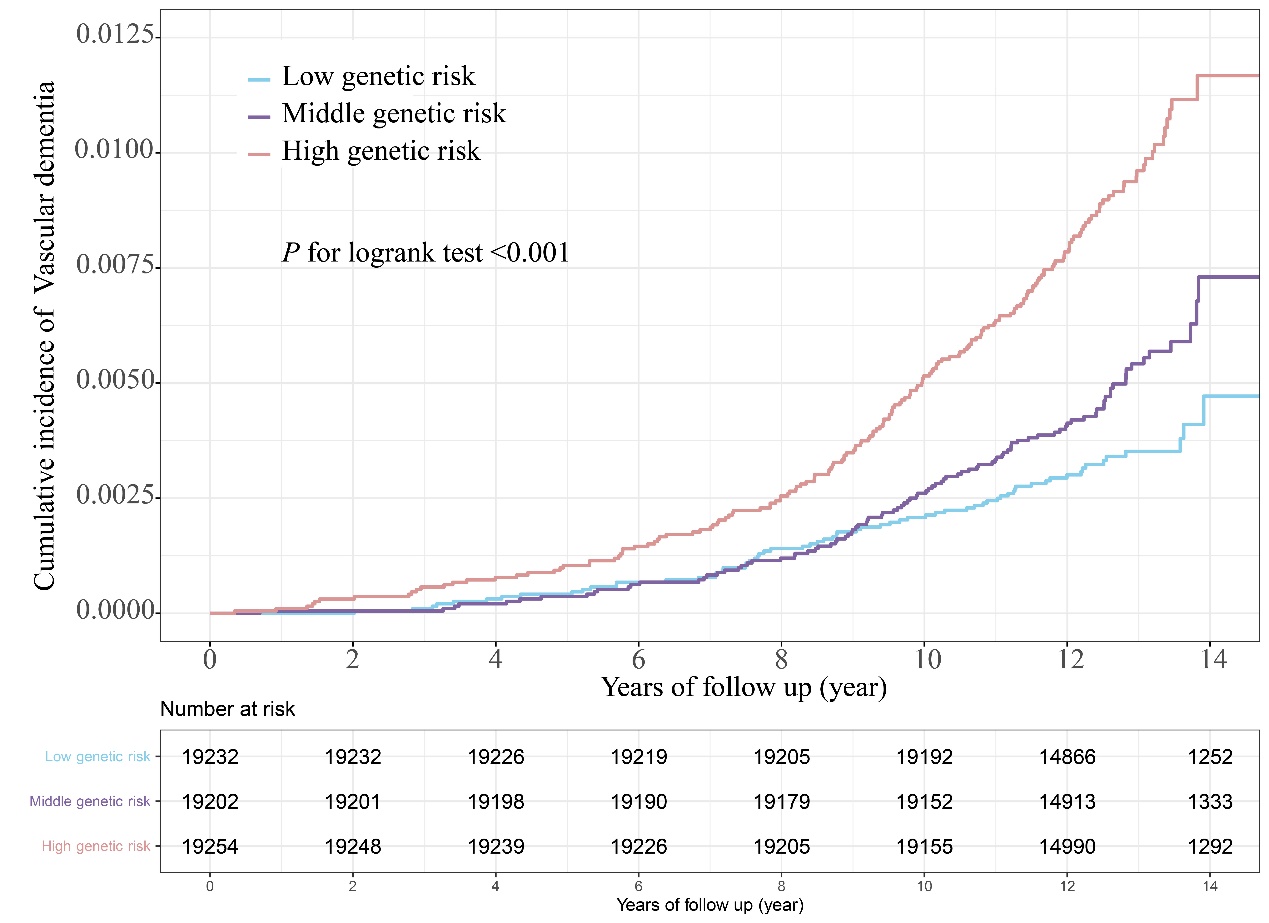


**Supplementary Figure 2.** The risk of incidence of dementia according to genetic risk
